# Detection of Monkeypox Virus DNA in Airport Wastewater, Rome, Italy

**DOI:** 10.3201/eid2901.221311

**Published:** 2023-01

**Authors:** Giuseppina La Rosa, Pamela Mancini, Carolina Veneri, Giusy Bonanno Ferraro, Luca Lucentini, Marcello Iaconelli, Elisabetta Suffredini

**Affiliations:** Istituto Superiore di Sanità, Rome, Italy

**Keywords:** Monkeypox, viruses, zoonoses, wastewater, airport, transportation hub, environmental surveillance, Rome, Italy

## Abstract

Environmental surveillance can be a complementary tool for detecting pathogens circulating in communities. We detected monkeypox virus DNA in wastewater from Italy’s largest airport by using real-time PCR assays targeting the G2R region and *F3L* and *N3R* genes and sequencing. Wastewater surveillance can be quickly adapted to investigate emerging threats.

Monkeypox virus (MPXV), a member of the family *Poxviridae*, causes monkeypox, a viral zoonosis detected in north Africa in the 1970s (*1*). MPXV can be transmitted between humans through contact with lesions, body fluids, respiratory droplets, and contaminated materials (*1*).

In May 2022, an epidemic of monkeypox in nonendemic regions outside Africa began receiving worldwide attention. On July 23, 2022, the World Health Organization declared monkeypox a public health emergency of international concern (*2*), and 24,973 monkeypox cases had been recognized in 45 countries throughout Europe by October 12, 2022 (*3*). 

Rapid identification of outbreaks and clusters is critical for infection control. Sewage surveillance has been recognized as a powerful tool for assessing the circulation of pathogens. After the European Union issued Recommendation 2021/472 (https://data.europa.eu/eli/reco/2021/472/oj), wastewater surveillance was successfully used to track SARS-CoV-2 and its variants across EU countries (*4*). Studies have demonstrated MPXV DNA sheds from saliva, feces, urine, semen, and skin lesions (*5*–*7*), suggesting that the viral genome could occur in wastewater. Various research groups involved in SARS-CoV-2 environmental surveillance extended their efforts to investigate MPXV DNA in wastewater. Studies from the Netherlands and western California, USA, have documented successful detection of MPXV DNA in sewage (*8*; M.K. Wolfe et al., unpub. data, https://doi.org/10.1101/2022.07.25.22278043). We investigated whether we could detect MPXV in wastewater in Italy.

## The Study

We targeted the wastewater treatment plant (WTP) of Italy’s largest airport, Fiumicino Airport, in Rome, which had ≈3,000,000 passengers/month during May–July 2022 (https://fiumicinoairport.com/statistics). This WTP has a global capacity of 4,000 m^3^ per day. We collected 24-hour composite wastewater samples twice a week during May 30–August 3, 2022, for a total of 20 samples.

Before viral concentration, we pretreated samples in a water bath at 56°C for 30 min to inactivate the virus and protect laboratory technicians, as per a previous study (*9*). We used a polyethylene glycol/sodium chloride precipitation protocol originally developed for SARS-CoV-2 environmental surveillance (*10*,*11*) but modified the protocol by increasing the initial wastewater volume to 90 mL (2 tubes of 45 mL) and eluting all the extracted nucleic acids in 50 µL of elution buffer supplied with the kit. We used NucliSens miniMAG (bioMérieux, https://www.biomerieux.com) semi-automatic extraction platform to extract nucleic acids. We used OneStep PCR Inhibitor Removal Kit (Zymo Research, https://www.zymoresearch.com) to purify DNA.

We used 3 different real-time PCR assays: 2 published in 2004 that target the *N3R* and *F3L* genes (*12*), and 1 developed in 2010 by the US Centers for Disease Control and Prevention, G2R_G generic real-time PCR assay (*13*), which targets the G2R region of the tumor necrosis factor receptor gene. After comparing primers and probes with sequences of the current outbreak, we noted mismatches in primers, probes, or both. Therefore, we designed and tested novel primers and probes that had 100% nucleotide identity with current outbreak sequences, then compared these with the original primers and probes (Table 1). We used MPXV (Slovenia ex Gran Canaria) DNA (European Virus Archive Global [EVAg]; https://www.european-virus-archive.com) as a control for testing primers and probes (Appendix). We further optimized the assays by evaluating different real-time PCR reagents and primer/probe concentrations (Appendix). We prepared reaction mixes in 25 μL by using TaqPath BactoPure Microbial Detection Master Mix (Thermo Fisher Scientific, https://www.thermofisher.com), 800 mmol of each primer, 500 nmol of the probe, and 5 μL of sample. Amplification conditions included an initial activation step at 95°C for 2 min and 50 cycles of 10 s at 95°C and 30 s at 60°C. We included 10-fold dilutions of the standardized EVAg MPXV DNA (range 740–0.74 copies/μL) in the runs as positive controls and for the rough estimation of viral loads. For each assay, we assessed the limit of detection at 50% (LOD_50_) on a pure target (i.e., EVAg MPXV DNA) and on MPXV DNA diluted in nucleic acids previously extracted from wastewater samples collected in Europe before monkeypox emerged.

**Table 1:** Primers and probes used to detect monkeypox virus DNA in airport wastewater, Rome, Italy*

PCR ID	Target	Primer name	Primer ID	Sequence, 5′ → 3′	Position†	Annealing temp. (°C)	Amplicon size	Ref.
1002	G2R	MPVX G F	2368	GGAAAATGTAAAGACAACGAATACAG	194459–84	60	90 bp	(*12*)
		MPVX G R	2369	GCTATCACATAATCTGGAAGCGTA	194525–48			
		MPVX G P	2370	FAM-AAGCCGTAATCTATG TTGTCTATCGTGTCC-BHQ1	194485–514			
1005	G2R	MPVX G F mod	2377	GGAAA**G**TGTAAAGACAACGAATACAG	194459–84	60	90 bp	(*12*)
		MPVX G R mod	2378	GCTATCACATAATCTG**A**AAGCGTA	194525–48			This study
		MPVX G P	2370	FAM-AAGCCGTAATCTATG TTGTCTATCGTGTCC-BHQ1	194485–514			
1003	*F3L*	F3L-F290	2371	CTCATTGATTTTTCGCGGGATA	46313–34	60	107 bp	(*11*)
		F3L-R396	2372	GACGATACTCCTCCTCGTTGGT	46398–419			
		F3Lp333S-MGB	2373	FAM-CATCAGAATCTGTAGGCCGT-MGBNFQ	46398–419			
1008	*F3L*	F3L-F290	2371	CTCATTGATTTTTCGCGGGATA	46313–34	60	107 bp	(*11*)
		F3L-R396 mod	2384	**A**ACGATACTCCTCCTCGTTGGT	46398–419			This study
		F3Lp333S-MGB	2373	FAM-CATCAGAATCTGTAGGCCGT-MGBNFQ	46398–419			
1004	*N3R*	N3R-F319	2374	AACAACCGTCCTACAATTAAACAACA	190641–66	60	139 bp	(*11*)
		N3R-R457	2375	CGCTATCGAACCATTTTTGTAGTCT	190755–79			
		N3Rp352S-MGB	2376	FAM-TATAACGGCGAAGAATATACT-MGBNFQ	190674–94			
1016	*N3R*	N3R-F319	2374	AACAACCGTCCTACAATTAAACAACA	190641–66	60	139 bp	(*11*)
		N3R-R457	2375	CGCTATCGAACCATTTTTGTAGTCT	190755–79			This study
		N3Rp352S-MGB mod	2381	FAM-TATAACGGCGA**C**GAATATACT-MGBNFQ	190674–94			
1006	G2R	G2R-1st cycle F	2379	ATAGCACCACATGCACCATC	194435–54	63	156 bp	This study
		G2R-1st cycle R	2380	AAAGGTATCCGAACCACACG	194590–71			
1005	G2R	MPVX G F mod	2377	GGAAAGTGTAAAGACAACGAATACAG	194459–84	61	90 bp	This study
		MPVX G R mod	2378	GCTATCACATAATCTGAAAGCGTA	194525–48			
1009	*F3L*	F3L-1st cycle F	2385	CAGGGTTAACACCTTTCCAA	46242–61	61	212 bp	This study
		F3L-1st cycle R	2386	TGATCTTCAACGTAGTGCTATGG	46453–31			
1008	*F3L*	F3L-F290	2371	CTCATTGATTTTTCGCGGGATA	46313–34	62	107 bp	(*11*)
		F3L-R396 mod	2384	AACGATACTCCTCCTCGTTGGT	46398–419			This study
1007	*N3R*	N3R-1st cycle F	2382	TCTATCTCGTTCATGGTCGGTAAT	190503–26	64	455 bp	This study
		N3R-1st cycle R	2383	CGCACTGTCTTATTCGCCATT	190957–37			
1004	*N3R*	N3R-F319	2374	AACAACCGTCCTACAATTAAACAACA	190641–66	64	139 bp	(*11*)
		N3R-R457	2375	CGCTATCGAACCATTTTTGTAGTCT	190755–79			

*Bold underlined text in bases represents modifications to the original primers and probes. ID, identification; F, forward; mod, modified; MPXV, monkeypox virus; R, reverse; ref., reference. †Position based on monkeypox virus reference isolate MPXV_USA_2022_MA001, complete genome, GenBank accession no. ON563414.

We designed nested PCR assays targeting the same regions as the real-time PCR assays to confirm results by amplicon sequencing using Primer3Plus software (https://www.primer3plus.com) (Table 1). We performed reactions by using 1 μL of 10 μmol primer and 2 μL of sample, and Platinum SuperFi II Green PCR Master Mix (Thermo Fisher Scientific) in a final volume of 25 μL. PCR amplicons on both strands were sequenced by Bio-Fab Research (https://www.biofabresearch.com). 

All real-time PCR assays successfully amplified the EVAg MPXV DNA. Compared with the original assay, the modified G2R_G assay showed a decrease in the average quantification cycle (Cq) values of 1.34 cycles (21.93 vs. 23.28), demonstrating a better performance. Therefore, we performed subsequent optimization activities and screening of wastewater samples by using the F3L and N3R assays as originally designed but modified the G2R_G assay for our study.

On pure MPXV DNA, the real-time F3L assay had an LOD_50_ of 0.21 copies/μL, the N3L assay had an LOD_50_ of 0.31 copies/μL, and G2R_G had an LOD_50_ of 0.21 copies/μL. For nucleic acids extracted from sewage samples spiked with MPXV, F3L had an LOD_50_ of 0.43 copies/μL and 2.16 copies/reaction, N3L had an LOD50 of 0.33 copies/μL and 1.65 copies/reaction, and G2R_G had an LOD_50_ of 0.31 copies/μL and 1.55 copies/reaction (Appendix).

Cq values ranged from 38.37–40.18 for 2 wastewater samples that tested positive by real-time PCR (Table 2), indicating relatively low DNA concentrations in the tested samples. Consensus sequences found 100% similarity by BLAST analysis between study sequences and MPXV strains available in GenBank (accession no. OX248696), thus confirming the presence of MPXV DNA.

**Table 2:** Wastewater sample results detecting monkeypox virus DNA in airport wastewater, Rome, Italy*

Sample ID	Collection date	Real-time RT-PCR (Cq values)				Nested RT-PCR		
		G2R	F3L	N3R		G2R	F3L	N3R
4419	2022 May 30	–	–	–		–	–	–
4420	2022 Jun 1	–	–	–		–	–	–
4421	2022 Jun 6	–	–	–		–	–	–
4422	2022 Jun 8	–	–	–		–	–	–
4444	2022 Jun 13	–	–	–		–	–	–
4445	2022 Jun 15	+ (40.18)	+ (39.59)			**+**	–	–
4453	2022 Jun 20	–	–	–		–	–	–
4454	2022 Jun 22	–	–	–		–	–	–
4460	2022 Jun 27	–	–	–		–	–	–
4461	2022 Jun 29	–	–	–		–	–	–
4474	2022 Jul 4	–	–	–		–	–	–
4475	2022 Jul 6	–	–	–		–	–	–
4476	2022 Jul 11	–	–	–		–	–	–
4477	2022 Jul 13	–	–	–		–	–	–
4478	2022 Jul 18	+ (38.37)	–	–		+	+	–
4479	2022 Jul 20	–	–	–		+	**+**	–
4480	2022 Jul 25	–	–	–		–	–	–
4481	2022 Jul 27	–	–	–		–	–	–
4482	2022 Aug 3	–	–	–		–	–	–
4483	2022 Aug 1	–	–	–		–	–	–

*Bold positive font (**+**) indicates sequence failure due to insufficient DNA target; amplification band of the expected length was confirmed by duplicate experiments. ID, identification; RT-PCR, reverse transcription PCR; –, negative; +, positive.

## Conclusions

A crucial aim of infectious disease surveillance is early detection of cases, outbreaks, and clusters, which is essential for disease control. We explored possible methods for monitoring MPXV through wastewater surveillance, a well-established complementary epidemiologic tool used successfully for viral infectious diseases, including SARS-CoV-2 and polio.

Monkeypox prevalence in the general population was low at the time of sample collection, only 20 cases had been detected in Italy as of May 30, 2022. Thus, to maximize the probability of positive samples among those collected, we tested wastewater samples from a large transportation hub, through which millions of persons travel to and from numerous countries. Because harmonized methods for detecting MPXV in wastewater are not yet available, we tested 3 different real-time PCR assays previously designed for clinical samples. We modified the assays by introducing changes in the primer and probe sequences to mitigate the effect of nucleotide mismatches. Among 20 samples, 3 tested positive for MPXV by real-time or nested PCR and sequencing.

In the next stage, we will test wastewater samples from WTPs enrolled in official SARS-CoV-2 environmental surveillance throughout Italy, to map the geographic distribution of MPXV in the country. Further research efforts should focus on elucidating how detection of viral DNA in sewage can be related to reported and confirmed cases. Factors affecting MPXV detection in wastewater also should be studied, including routes and duration of virus shedding by infected persons, environmental persistence, and analytical sensitivity of the methods used (*14*). 

In conclusion, we adapted SARS-CoV-2 wastewater surveillance for MPXV detection in a large airport WTP. Our methods can be applied to wastewater-based epidemiology for monkeypox outbreaks and provides basic tools, including analytic methods. Wastewater surveillance can be rapidly adapted to detect emerging threats, including monkeypox.

Appendix Additional information on detection of monkeypox virus DNA in airport wastewater, Rome, Italy.
