# Supplementary material for: Detection of Monkeypox Virus DNA in Airport Wastewater, Rome, Italy
Source: Emerg Infect Dis. 2023 Jan;29(1):193–6. doi: 10.3201/eid2901.221311 (PMC9796218; doi:10.3201/eid2901.221311)
Supplement: Appendix — Additional information on detection of monkeypox virus DNA in airport wastewater, Rome, Italy. [file 22-1311-Techapp-s1.pdf]

Article DOI: https://doi.org/10.3201/eid2901.221311

Detection of Monkeypox Virus DNA in Airport Wastewater, Rome, Italy

Appendix

Comparison of Primers and Probe Sets

To select the primer and probe sets providing better real-time PCR results, a 10^{-2} dilution of the Monkeypox DNA Slovenia ex Gran Canaria, Ref-SKU: 005N-04716 provided by EVAg was tested as control, using PCR IDs 1002, 1003, 1004, 1005, 1008, 1016 (Table 1).

For the sake of comparison, all primer/probe sets were assayed in the same conditions: real-time reactions were prepared in 25 μL volume using the TaqPath BactoPure Microbial Detection Master Mix (Thermo Fisher Scientific, https://www.thermofisher.com), 500 mmol of each primer, 250 nmol of each probe, and 5 μL of sample DNA. Amplification conditions included an initial activation at 95°C for 2 min, and 45 cycles of 10 s at 95°C and 30 s at 60°C. Each reaction was run in triplicate on a QuantStudio 12K Flex (Applied Biosystems-Thermo Fisher) (Appendix Table 1). The 3 primer/probe sets with the lowest quantification cycle (Cq) value were selected for further optimization: PCR identification nos. 1003 (F3L), 1004 (N3R), and 1005 (G2R).

Standardization of Monkeypox DNA provided by the European Virus Archive Global (EVAg)

To standardize material for the optimization and performance characterization of the selected real-time PCR, we quantified used Monkeypox virus (Slovenia ex Gran Canaria) DNA batch 06.06.2022 (European Virus Archive Global [EVAg]; https://www.european-virus-archive.com) the Monkeypox DNA (Monkeypox virus, 2022, Slovenia ex Gran Canaria, Ref. 005V-04714) by droplet digital PCR (ddPCR).

Tenfold dilutions of the DNA stock were prepared in molecular grade Tris-EDTA buffer (10 mM Tris, 1 mM EDTA) pH 8.0 (Sigma-Aldrich, https://www.sigmaaldrich.com). DNA

dilutions of 10^{-2}, 10^{-3}, 10^{-4} and 10^{-5} were tested in quadruplicate by ddPCR by using the QX200 system (Bio-Rad, https://www.bio-rad.com) and the ddPCR Supermix for probes kit without deoxyuridine triphosphate (dUTP) (BioRad). The reaction mixture included: 10 µL ddPCR supermix, primers 500 nmol, probes 250 nmol, and nuclease-free water to a final volume of 20 µL. Primers and probes were those described for PCR nos. 1003, 1004, and 1005. Droplets were generated as recommended by the manufacturer and amplification was performed on a 9600 GenAmp thermalcycler (Applied BioSystems) as follows: 95°C for 10 min, followed by 94°C for 30 s and 60°C for 60 s (40 cycles), and by a final stage at 98°C for 10 min. Results were acquired using the Bio-Rad QX200 Droplet Reader and QX Manager Standard Edition version 1.2 to provide absolute quantification of the target sequence (Appendix Table 2).

Because 2 copies of the G2R region are in MPXV genome due to its location at the ITR terminal regions, only the results of F3L and N3R were taken into account for the quantification of viral genome copies (copies/µL) of the Monkeypox Slovenia ex Gran Canaria, Ref-SKU: 005N-04716 provided by EVAg. Reference value was defined as the average of the values obtained for the 2 targets: 738,419 copies/µL = 7.4×10^5 copies/µL.

Optimization of Real-Time PCR Assays 1003 (F3L), 1004 (N3R), and 1005 (G2R) conditions

Testing of Different Real-Time Reagents

Based on previous experiences, the choice of real-time reagents may affect the efficiency of target virus detection in wastewater samples, due to different sensitivity of polymerases to factors as environmental inhibitors or supercoiling of target sequences. To select the most efficient reagents for Monkeypox DNA detection in wastewater, the standardized EVAg Monkeypox DNA was diluted in a 1:100 proportion in nucleic acids extracted from wastewater samples collected from urban WTPs in a period preceding the emergence of Monkeypox virus in Italy (November and December 2021).

Samples were then tested with the 3 PCR assays by using the following reagents: AgPath-ID One-Step RT-PCR Reagents (Applied Biosystems) for PCR 1003, TaqPath BactoPure Microbial Detection Master Mix (Thermo Fisher Scientific) for PCR 1004, and TaqMan Fast Advanced Master Mix (Thermo Fisher Scientific) for PCR 1005.

All reactions were prepared in 25 µL volume using 500 mmol of each primer, 250 nmol of probe and 5 µL of sample DNA. AgPath-ID reactions included 1 µL/reaction of enzyme mix and 1.67 µL of detection enhancer. Reactions were run on a QuantStudio 12K Flex (Applied Biosystems).

The following amplification conditions were used to obtain Cq values (Appendix Table 3). For AgPath-ID One-Step RT-PCR Reagents, an initial reverse transcription inactivation step at 95°C for 5 min, followed by 45 cycles of 10 s at 95°C and 30 s at 60°C. For TaqPath BactoPure Microbial Detection Master Mix, an initial activation step at 95°C for 2 min, followed by 45 cycles of 10 s at 95°C and 30 s at 60°C. For TaqMan Fast Advanced Master Mix, an initial hold at 50°C for 2 min, followed by an activation step at 95°C for 20 s, and by 45 cycles of 10 s at 95°C and 30 s at 60°C. Because no significant difference was detected among the performance of the different reagents, we selected TaqPath BactoPure Microbial Detection Master Mix, because it required the least preparation time.

Primer/Probe Concentrations

Optimization of primer/probe concentrations was performed by testing the following concentrations in their different combinations, for each PCR assay (IDs 1003, 1004, and 1005): forward primer at 200 nmol, 500 nmol, 800 nmol; reverse primer at 200 nmol, 500 nmol, 800 nmol; and probe at 100 nmol, 250 nmol, 500 nmol. All reactions were prepared in 25 µL volume by using 5 µL of sample DNA (10^{-4} dilution of the standardized EVAg Monkeypox DNA) and the TaqPath BactoPure Microbial Detection Master Mix. Amplification conditions included an initial activation step at 95°C for 2 min, followed by 45 cycles of 10 s at 95°C and 30 s at 60°C. Reactions were run on a QuantStudio 12K Flex (Applied Biosystems), and graphically summarized results (Appendix Figure).

Based on Cq values, in all PCR assays, better amplifications were achieved at a probe concentration of 500 nmol and slightly better results were obtained with a primer concentration of 800 nmol. Although, in the primer, the differences with other concentrations were minimal (often >1 ΔCq). Therefore, concentrations of 500 nmol of probe and 800 nmol of primers were used for the analysis of the environmental samples.

Assessment of LOD_{50} for real-time PCR ID 1003 (F3L), 1004 (N3R) and 1005 (G2R)

LOD_{50} on Pure Target (Monkeypox DNA Diluted in TE buffer)

To assess the sensitivity of the real-time PCR assays used in the study, the LOD_{50} of each reaction was calculated according to Wilrich and Wilrich (https://www.wiwiss.fu-berlin.de/fachbereich/vwl/iso/ehemalige/wilrich/index.html). We prepared 2-fold dilutions of the standardized EVAg Monkeypox DNA in molecular grade TE buffer pH 8.0 starting from the 10^{-5} dilution (7.4 copies/µL). Each dilution was tested in 8 replicates with PCR 1003 (F3L), PCR 1004 (N3R), and PCR 1005 (G2R) by using the optimized reaction conditions (Appendix Table 4).

LOD_{50} on Target in Wastewater (Monkeypox DNA Diluted in Nucleic Acid Extracted from Wastewater Samples)

To assess the sensitivity of the real-time PCR assays in the condition of use (i.e., detecting monkeypox virus in wastewater samples), the LOD_{50} of each reaction was also calculated, using the same approach described above, by testing the target monkeypox DNA diluted in nucleic acid extracted from wastewater samples, which include potential inhibitors of the polymerization reaction.

We prepared 2-fold dilutions of the standardized EVAg Monkeypox DNA in molecular grade TE buffer pH 8.0 starting from the 10^{-4} dilution (74 copies/µL). Each dilution was then used to spike 1:10 proportion nucleic acid extracted from wastewater samples collected from urban wastewater treatment plants during November–December 2021, prior to emergence of monkeypox virus in Italy. Spiked samples were tested in 8 replicates with PCR 1003 (F3L), PCR 1004 (N3R), and PCR 1005 (G2R), using the optimized reaction conditions (Appendix Table 5).

Appendix Table 1. Primers used to detect monkeypox virus DNA in airport wastewater, Rome, Italy*

PCR ID	Target	Primer name	Primer ID	Average Cq	SD
1002	G2R	MPVX G F	2368	23.28†	0.55
		MPVX G R	2369		
		MPVX G P	2370		
1005	G2R	MPVX G F mod	2377	21.93	0.25
		MPVX G R mod	2378		
		MPVX G P	2370		
1003	F3L	F3L-F290	2371	22.95	0.31
		F3L-R396	2372		
		F3Lp333S-MGB	2373		
1008	F3L	F3L-F290	2371	23.56	0.25
		F3L-R396 mod	2384		
		F3Lp333S-MGB	2373		
1004	N3R	N3R-F319	2374	21.76	0.16
		N3R-R457	2375		
		N3Rp352S-MGB	2376		
1016	N3R	N3R-F319	2374	26.69†	0.43
		N3R-R457	2375		
		N3Rp352S-MGB mod	2381		

*Cq, quantification cycle; ID, identification; F, forward; mod, modified; MPXV, monkeypox virus; P, probe; R, reverse.
†Duplicate reaction due to run setting failure.

Appendix Table 2. Control DNA used in 3 PCR assays to detect monkeypox virus DNA in airport wastewater, Rome, Italy*

Dilution	PCR 1005, G2R	PCR 1003, F3L	PCR 1004, N3R
10^{-2}	NA†	7,665.25	6,973.25
10^{-3}	1,171.5	751.75	739.25
10^{-4}	120	76.5	78.75
10^{-5}	16	7.25	6.75
Weighted average	13,238.33	7,520.69	7,247.69
Stock DNA, copies/µL	1.3×10^6	7.5×10^5	7.2×10^5

*Controls expressed as copies/µL. NA, not achieved.
†Suboptimal separation of positive and negative events

Appendix Table 3. Reagents used in 3 PCR assays to detect monkeypox virus DNA in airport wastewater, Rome, Italy*

Reagent	PCR 1005, G2R	PCR 1003, F3L	PCR 1004, N3R
AgPath-ID One-Step RT-PCR	21.71	23.07	21.60
TaqPath BactoPure Microbial Detection Master Mix†	21.90	23.18	21.91
TaqMan Fast Advanced Master Mix	22.20	22.60	21.78

*Reagents available from Thermo Fisher Scientific, https://www.thermofisher.com.

Appendix Table 4. Target concentrations and limits of detection used to detect monkeypox virus DNA in airport wastewater, Rome, Italy*

Concentration and limit of detection	PCR 1005, G2R	PCR 1003, F3L	PCR 1004, N3R
Expected target concentration, copies/µL			
3.7	8/8	8/8	8/8
1.85	8/8	8/8	8/8
0.925	7/8	8/8	8/8
0.4625	7/8	5/8	7/8
0.23125	4/8	2/8	3/8
0.115625	3/8	1/8	2/8
Limit of detection			
LOD_{50} copies/µL	0.213	0.306	0.206
LOD_{95} copies/µL	0.923	1.323	0.890
LOD_{50} copies/reaction	1.065	1.530	1.030
LOD_{95} copies/reaction	4.615	6.615	4.450

*Results are expressed as no. positive/no. analytic replicates. LOD_{50}, limit of detection for which the probability of detection is 50%; LOD_{95}, limit of detection for which the probability of detection is 95%.

Appendix Table 5. Target concentrations and limits of detection used to detect monkeypox virus DNA in airport wastewater, Rome, Italy*

Concentration and limit of detection	PCR 1005, G2R	PCR 1003, F3L	PCR 1004, N3R
Expected target concentration, copies/μL			
7.4	8/8	8/8	8/8
3.7	8/8	8/8	8/8
1.85	8/8	8/8	8/8
0.925	6/8	7/8	7/8
0.4625	3/8	6/8	5/8
0.23125	3/8	1/8	3/8
Limit of detection			
LOD_{50}, copies/μL	0.432	0.330	0.309
LOD_{95}, copies/μL	1.868	1.427	1.336
LOD_{50}, copies/reaction	2.160	1.650	1.545
LOD_{95}, copies/reaction	9.340	7.135	6.680

*Results are expressed as no. positive/no. analytic replicates. LOD_{50}, limit of detection for which the probability of detection is 50%; LOD_{95}, limit of detection for which the probability of detection is 95%.

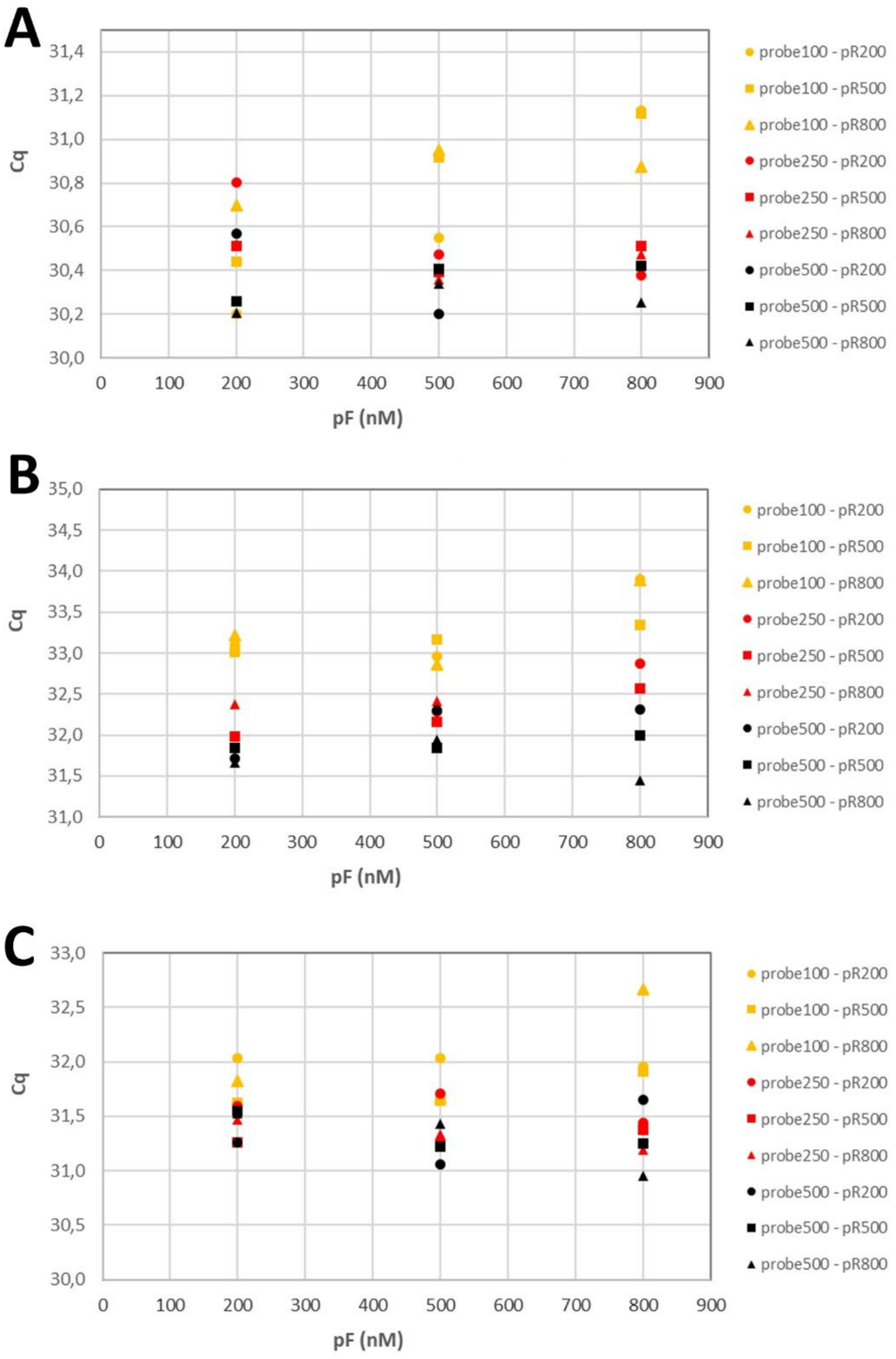

Appendix Figure. Optimization of primer/probe concentrations used to detect monkeypox virus DNA in airport wastewater, Rome, Italy. Optimization was performed by testing concentrations in their different combinations for each of 3 PCR assays. A) PCR 1003 for F3L; B) PCR 1004 for N3R; and C) PCR 1005 for G2R.
